# Unified Simulation Platform for Interference Microscopy

**DOI:** 10.1021/acsphotonics.4c00621

**Published:** 2024-06-17

**Authors:** Felix Hitzelhammer, Anežka Dostálová, Ilia Zykov, Barbara Platzer, Clara Conrad-Billroth, Thomas Juffmann, Ulrich Hohenester

**Affiliations:** † Institute of Physics, University of Graz Universitätsplatz 5, 8010 Graz, Austria; ‡ University of Vienna, Faculty of Physics, VCQ, 1090 Vienna, Austria; § University of Vienna, Max Perutz Laboratories, Department of Structural and Computational Biology, 1030 Vienna, Austria; ∥ Department of Optics Faculty of Science, Palacký University, 17. Listopadu 12, 77900 Olomouc, Czech Republic

**Keywords:** interference microscopy, coherent bright-field microscopy, simulation, particle tracking, nanosensing

## Abstract

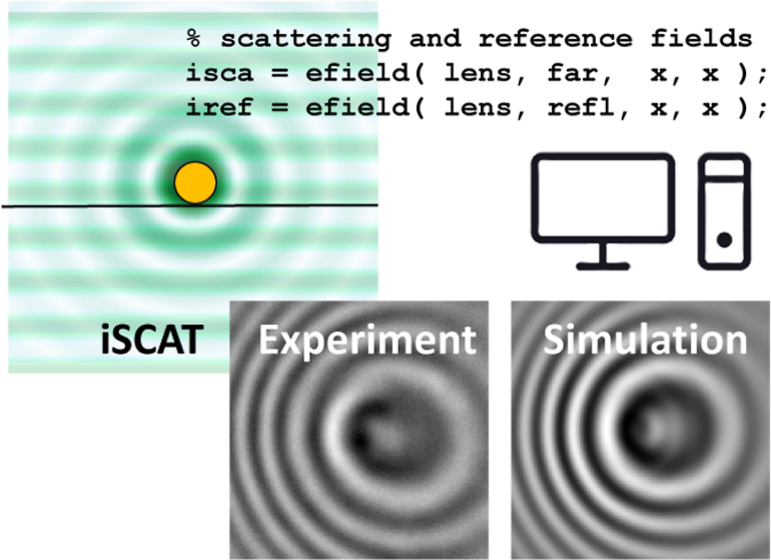

Interferometric scattering microscopy is a powerful technique that enables various applications, such as mass photometry and particle tracking. Here, we present a numerical toolbox to simulate images obtained in interferometric scattering, coherent bright-field, and dark-field microscopies. The scattered fields are calculated using a boundary element method, facilitating the simulation of arbitrary sample geometries and substrate layer structures. A fully vectorial model is used for simulating the imaging setup. We demonstrate excellent agreement between our simulations and experiments for different shapes of scatterers and excitation angles. Notably, for angles near the Brewster angle, we observe a contrast enhancement which may be beneficial for nanosensing applications. The software is available as a matlab toolbox.

## Introduction

Interferometric scattering microscopy (iscat) allows for the detection of nanoparticles with unprecedented sensitivity at high spatiotemporal resolution by exploiting the interference between scattered light from a sample and a reference beam. The technique has rapidly developed in recent years, finding numerous applications in biosciences and medicine, including the characterization of single proteins,^1^ and the tracking of metal nanoparticles on microsecond time scales.^2^ Related techniques such as coherent bright-field imaging (cobri)^3,4^ and dark-field imaging can in principle yield similar sensitivities.^5^

After initial proof-of-concept experiments, one of the primary goals has been to push the sensitivity limits to detect smaller and smaller particles, eventually enabling high-precision mass photometry of proteins.^1^ For such applications, iscat images can be accurately simulated using analytical models.^5,6^ These models have been shown to accurately predict iscat contrast levels and provide insights into the effects of aberrations, the z-dependence of the expected contrast, and the shot-noise limited maximally achievable localization and mass estimation precision. However, they typically only consider a single-point dipole, or a spherical scatterer, inside a homogeneous medium.

Interferometric imaging techniques are often used for tracking particles in cellular environments^2^ or for sensing on top of structured substrates.^7^ Furthermore, it has been demonstrated that high-precision quantitative agreement between experiment and simulation can only be achieved when the roughness of coverslips^8^ and plasmonic enhancements^9^ are taken into account. Such complex geometries are difficult to simulate analytically and therefore require numerical approaches.^6,9,10^

Here, we simulate interferometric microscopy images using the boundary element method (bem)^11,12^ and its implementation within the nanobem toolbox.^13,14^ Details on the software development are provided in the Supporting Information. Within numeric constraints, arbitrary scattering geometries can be implemented concerning nanoparticle shape, nanoparticle distribution, substrate layer structure, illumination direction, and illumination polarization. The scattered far fields are calculated and propagated to the camera using a fully vectorial imaging model based on the Richards–Wolf approach.^15^ This enables the simulation of high numeric aperture (NA) setups, including the most common iscat geometries with both full-field or scanning excitation as well as the transmissive full-field approach, which we call cobri in the following. We simulate iscat images for off-axis illumination and demonstrate a contrast enhancement and tunability close to the Brewster angle. In addition, we find excellent agreement between our simulated results and experimental iscat data for a gold nanosphere and a silver nanocube.

We have organized our paper as follows. In the Theory section, we present the theory underlying interference microscopy, the bem approach, and the Richards–Wolf approach for imaging. Selected simulation results and a comparison with experiments are given in the Results section. Finally, we provide a brief summary. The simulation software and a short user manual can be found in the Supporting Information.

## Theory

### Outline of the Problem

**Figure 1:**
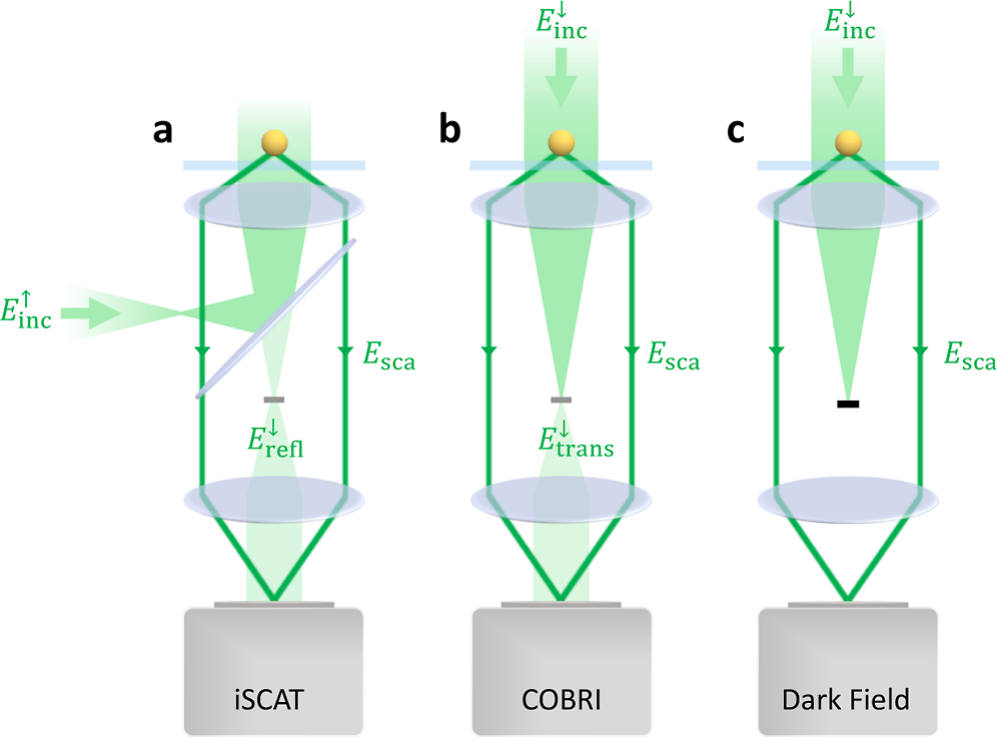
Sketch of microscopy configurations. (a) In iscat, the interference image is formed between the (possibly attenuated) field reflected at the coverslip and the fields scattered by the nanoparticle. (b) In cobri, the interference image between the (possibly attenuated) transmitted incoming and scattered fields is measured. (c) In dark-field microscopy, only the scattered fields are imaged. Figure adapted from Dong et al.^5^

We consider the situation depicted in Figure 1 where a nanoparticle located above a substrate is excited by a monochromatic incoming laser, and the interference between the (reflected or transmitted) incoming light and the light scattered by the nanoparticle is imaged through a microscopy setup. In iscat, the nanoparticle is excited from below, and the total incoming field is the sum of the primary field ***E***_inc_^↑^ impinging from below on the substrate and the secondary fields resulting from reflection and transmission at the interface
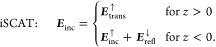
1The superscript arrows indicate the propagation direction of the fields, given with respect to the *z*-direction. In cobri, the primary field ***E***_inc_^↓^ impinges on the interface from above, and the total incoming field is again the sum of primary and secondary fields
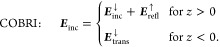
2eqs 1 and 2 can be generalized for stratified media, i.e., vertically stacked materials with multiple interfaces, supplementing the primary incoming fields by the proper fields inside the various media. See eqs 11 and 12 for a detailed discussion of how to compute these fields. In the following, we will assume that ***E***_inc_ is known.

In the presence of a nanoparticle, the incoming fields become scattered by the nanoparticle, and the total field is the sum of incoming and scattered fields
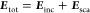
3

Far away from the nanoparticle and the interfaces of the stratified medium, the fields pass through the lenses of an imaging system, as will be described in more detail below and are transformed according to
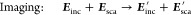


The image on the camera is then proportional to the field intensity, and we get
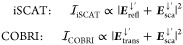
4

When describing iscat or cobri theoretically, usually the different image formation steps are modeled in a consecutive order and with various degrees of sophistication. For incoming plane waves and a substrate, the reflected and transmitted waves can be obtained with the usual Fresnel coefficients.^16^ The nanoparticle is usually modeled as a polarizable particle with homogeneous material parameters, and the light scattered by the nanoparticle is approximated by the emission of a point dipole.^6^ Finally, the imaging of the secondary and scattered fields is described through the Richards–Wolf approach,^15^ which we will discuss in more detail below. While for small nanoparticles the agreement between the results of such an approach and experiment is usually very good,^5,6^ deviations might occur for larger, coated, or coupled nanoparticles, as well as for tilted or structured incoming light fields. In such cases, a general Maxwell solver should be used that allows computing the scattered electromagnetic fields for arbitrary incoming fields and nanoparticle geometries.

### Ingredients of Interference Microscopy Toolbox

In this work, we employ the open-source toolbox nanobem that is based on a boundary element method (bem) approach and is implemented in matlab.^13,14^ To render the toolbox suitable for the simulation of various interference microscopy techniques, additional features are required, which are listed in Table 1. We consider a single plane wave or a plane wave decomposition of focused laser fields for the incoming fields and a far-field representation for the scattered fields. For both types of field representation, we have implemented imaging transformations based on the Richards–Wolf approach.^15^ To allow for the rotation of the optical axis and the shift of the focus position, we require additional operations that enable the rotation and shifting of the field representations. These operations are usually encapsulated in the toolbox functions and are discussed at some length in the Supporting Information.

**Table 1:** Field Manipulations Needed to Render the nanobem Toolbox Suitable for the Simulation of Interference Microscopy^a^

Object	transformation	description
rotation matrix R	*R****F***, *Rr̂*	rotate far-field amplitude and propagation direction
	*R***ϵ**, *R****k***	rotate field and wavevector for plane wave decomposition
shift vector ***r***_0_	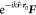	shift coordinate system of far-field expansion from ***r*** → ***r*** + ***r***_0_
	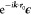	shift coordinate system of plane wave decomposition
imaging lenses	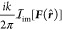	image far fields using the Richards–Wolf approach
	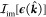	image plane wave decomposition using the Richards–Wolf approach

a For the representation of the electromagnetic fields, we consider far fields and plane wave decompositions. For details, see text.

For the incoming fields, we use a plane wave decomposition
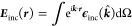
5where  is the field amplitude for the propagation direction ***k̂*** and *d*Ω denotes the integration over the unit sphere. With this, we can also describe a plane wave with polarization **ϵ_0_** and propagation direction  by setting , where θ_0_ and ϕ_0_ are the polar and azimuthal angles of the light propagation direction, respectively. For the scattered fields, we use a far-field representation in terms of outgoing spherical waves modulated by the far-field amplitude , see eq 3.5 of ref (12)
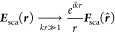
6

In our simulation approach, primary incoming fields are specified, which are typically either plane waves  or the focal fields of an incoming laser field, see eq (3.47) of ref (16)
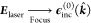


See the Supporting Information for more details of how to obtain these fields. In a second step, the fields are used as an input for the bem solver, which computes the secondary incoming fields **ϵ**_inc_^(1)^ and the scattered far fields
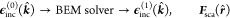
which are finally submitted to the imaging procedure of the Richards–Wolf approach. We then obtain the iscat or cobri images through
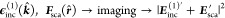


### BEM in a Nutshell

In bem, nanoparticles with homogeneous material parameters, which are separated by abrupt interfaces from the embedding medium, are considered. The shape of the particles can be chosen arbitrarily, and coupled or coated particles modeled accurately. Whether the restriction of homogeneous media applies to a given system under study must be decided on a case-by-case basis. In the following, we briefly introduce the methodology of bem, more details can be found in the specialized literature.^11,12^

Consider a nanoparticle embedded in a background medium with the permittivity and permeability functions ε, μ, respectively. The Stratton-Chu^17^ or representation formula, see eq 5.37 of ref (12), then relates the scattered electric field at position ***r*** inside the background medium to the tangential electromagnetic fields at the particle boundary via
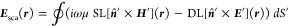
7

The integration extends over the nanoparticle boundary, and ***n****^* is the surface normal pointing away from the particle. The single-and double-layer potentials SL and DL, respectively, are related to the dyadic Green’s tensor for the stratified medium and describe how the tangential fields propagate from the nanoparticle boundary at position ***s***′ to ***r***.

The representation formula of eq 7 can be used in two different ways. First, by letting ***r*** → ***s*** approach a position on the boundary, we obtain the so-called Calderon identity, which relates the electric field ***E***(***s***) on the boundary (left-hand side) to an integral over the tangential boundary fields (right-hand side). Combining the Calderon identities for the electromagnetic fields outside and inside the particle with the usual Maxwell boundary conditions of continuous tangential electromagnetic fields, we obtain an expression that allows computing the tangential boundary fields for a given incoming field. This expression forms the heart of computational bem solvers, see eq 11.41 of ref (12). Second, knowing the tangential fields on the boundary, we can use eq 7 to compute the fields away from the boundary. Because the layer potentials SL and DL describe the field propagation in the stratified medium, we can perform the far-field limit analytically to arrive at
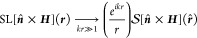
8with a similar expression for the double-layer potential.^12^ Here,  is a function that depends on the tangential boundary fields and the propagation directions ***r****^* only.

**Figure 2:**
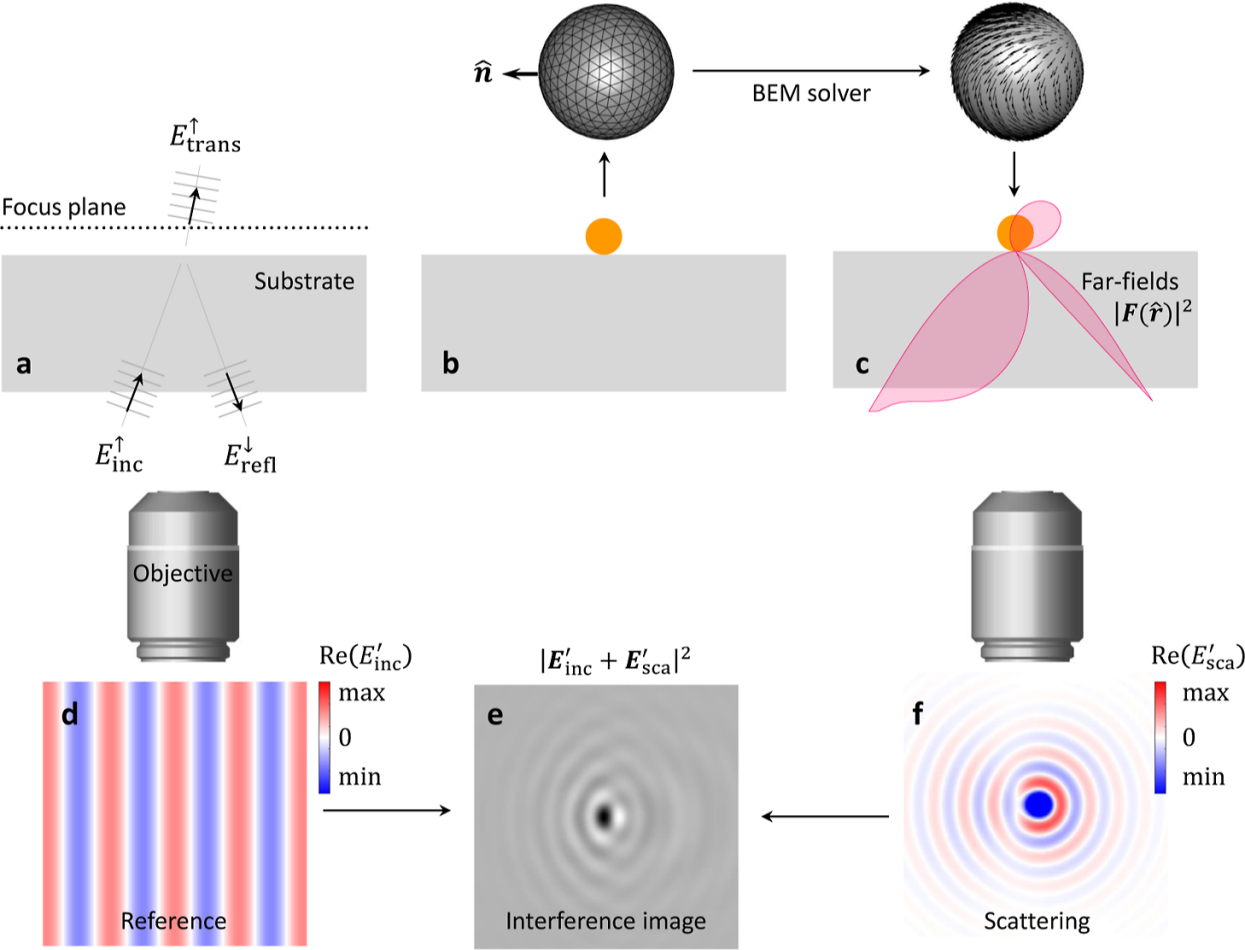
(a) In our simulations, we consider a substrate or stratified medium that is excited by incoming fields. (b) Optical response of an additional nanoparticle, here a gold nanosphere, is obtained from a bem solver. The boundary of the nanoparticle is discretized through triangular boundary elements, and by solving the bem working equations, the tangential electromagnetic fields at the particle boundary, which fully characterize the solution, are obtained. (c) From the bem solutions, we can compute the optical far fields, which are imaged using the Richards–Wolf approach. Through the superposition of the imaged (d) reference and (f) scattering fields, we finally obtain the (e) interference image that can be directly compared with results of iscat experiments.

Figure 2 shows the schematics of a typical bem simulation. (a) We start by defining a reference structure, here a substrate, and the incoming fields, here a plane wave impinging from below on the interface. Because of the contrast in refractive indices, part of the wave is reflected and transmitted at the interface. (b) The modification of the electromagnetic fields in the presence of a nanoparticle, here a gold nanosphere, is accounted for through the bem solver: the boundary of the nanoparticle is discretized in terms of triangular boundary elements, and by solving the bem working equation, see eq 9 of ref (14), the tangential boundary fields that fully characterize the solution of the problem under study are obtained. (c) By submitting the far fields to an imaging transformation, as described in more detail in the following, we obtain the fields in the image plane of the objective. The superposition of the (f) scattered and (d) reference (reflected) fields allows us to finally compute the (e) interference image, see eq 4, which can be directly compared with the results of iscat or cobri experiments.

### Richards–Wolf Approach

**Figure 3:**
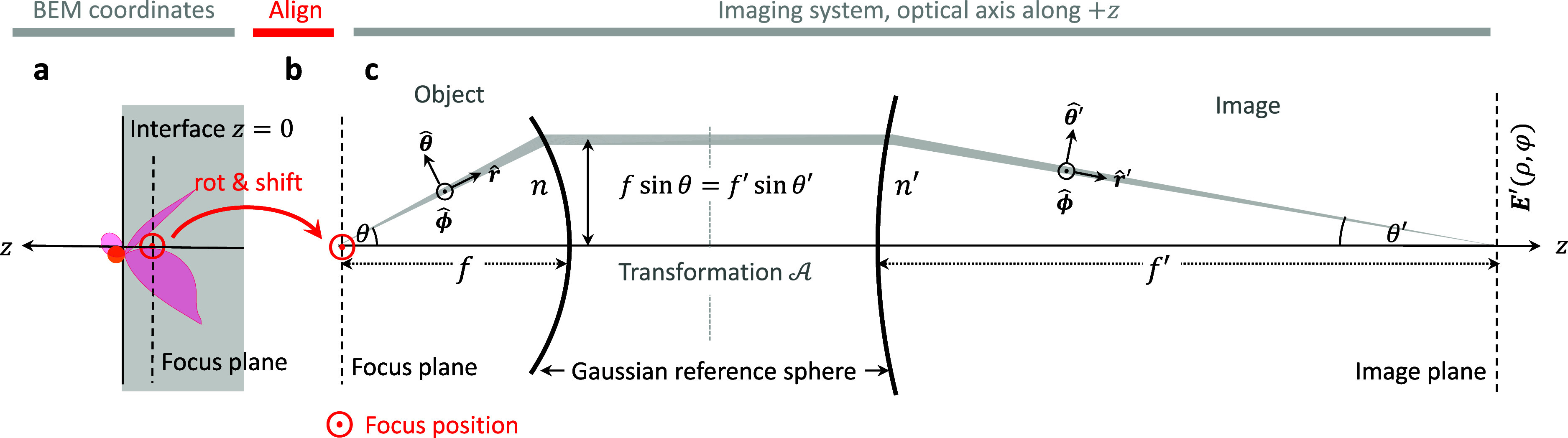
Imaging through an aplanatic lens. (a) bem simulation using a coordinate system where *z* = 0 coincides with the interface of the substrate. (b) Through a rotation and shift of the fields, we transform them to the (c) coordinate system for imaging, with the focus point at *z* = 0 and the optical axis pointing along +*z*. The far fields emanating from the focus position are imaged in the image plane. In the Richards–Wolf approach, the light rays are redirected at the Gaussian reference spheres, and we additionally consider the conservation of energy transported by the waves. We use unprimed spherical coordinates on the object side and primed coordinates on the image side. Details can be found in the text. A transformation  in the back focal plane between the two reference spheres allows us to manipulate the fields and mimic, e.g., the effect of a quarter wave plate.

The working principle of the Richards–Wolf approach for imaging has been described at length elsewhere.^6,12,15,16,18^ Here, we only sketch the main steps. The approach requires the secondary incoming fields and the scattered far fields as input and gives the imaged fields as output. The imaging of the objective is modeled by two Gaussian reference spheres with radii *f* and *f*′, as shown in Figure 3c. On the object side, the fields emanate from the focus point and become redirected at the Gaussian reference spheres. As a result of these operations, we obtain an image of the fields in the focal plane that is magnified by a factor of
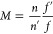
9where the refractive indices on the object and image side are indicated with unprimed and primed symbols *n*, *n*′, respectively. The same convention of unprimed (object) and primed (image) symbols is adopted for all other quantities, such as coordinates or vectors.

Let us pause for a moment and clarify a point that can cause some confusion, namely, the location of the focus spot. Suppose first that in Figure 3c, the space left to the first reference sphere (with focus *f*) is filled with a homogeneous medium with refractive index *n*, for which the microscope objective was designed, e.g., oil, and ***r***_foc_ is the focus spot that forms the origin of the coordinate system of the imaging system. If we send a light beam propagating parallel to the optical axis in the opposite direction back to the first reference sphere, it will be focused at ***r***_foc_. When replacing the homogeneous medium with a stratified medium, where the medium closest to the reference sphere has the refractive index *n* again, we keep ***r***_foc_ fixed. However, a light beam propagating back through the reference sphere does not necessarily focus at ***r***_foc_ but might be diffracted because of the stratified medium. In this respect, ***r***_foc_ is a hypothetical focus point that is needed in our theoretical approach but might be a point of no particular significance in the experiment. As shown in Figure 3a, the coordinate systems of the bem simulation and the imaging system can also differ. In bem, *z* = 0 is usually defined as the upper interface position of the stratified medium. In contrast, for the imaging system the center is located at the focus point, and the optical axis is assumed to point in the + *z*-direction. To transform from one coordinate system to the other, we have to rotate and shift the fields, as discussed in more detail in the Supporting Information. Shifting displaces the focus point with respect to the bem coordinate system, corresponding to the defocusing procedure conveniently employed in iscat experiments.

In the Richards–Wolf approach, we trace the fields through the optical setup and ensure energy conservation. For simplicity, we consider lenses with antireflecting coatings, thus neglecting back-reflections, and perfect imaging properties. In principle, both approximations can be easily lifted, see, for instance, Section 3.6 of ref (12) or eq 14 of ref (6). On the object side, we introduce a spherical coordinate system with the unit vectors **θ̂**, **ϕ̂**, ***r*^**. Since the far fields propagate in the direction ***r*^**, the transverse electromagnetic fields only have components in the θ, ϕ directions. The ϕ component of the electric field has a te character and thus keeps its orientation when propagating through the optical system, see also Figure 3c. In contrast, the θ component has a tm character and is rotated from the **θ̂** to the  direction. With the additional requirement of the conservation of energy transported by the waves, see eq 3.30 of ref (12), we are then led to a relation between the electric fields on the object and image side via
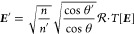
10with the rotation matrix  that accounts for the rotation of the unit vectors from the object to the image side and the transformation *T*[***E***] that accounts for the aforementioned change of coordinate systems. In the last step, we assume *f*′ ≫ *f* and perform the paraxial approximation by setting cos θ′ ≈ 1, such that  approximately points in the *z*-direction. With this, we are led to the Richards–Wolf integral
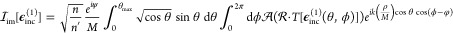
11where θ_max_ is the opening angle of the first reference sphere, which is related to the numerical aperture of the lens via *n* sin θ_max_ = NA and ψ is a phase that is of no importance for our present concern.  is an optional function that can be used to transform the fields in the back focal plane, see Figure 3. It could, for example, be used to model for the effect of a quarter wave plate,^19^ an attenuator for contrast enhancement, or a beam block for dark-field microscopy. For details, see the Supporting Information. Expressing the image coordinates *x*′, *y*′ in polar coordinates ρ, φ, we get for the imaged secondary fields . Similarly, for the imaging of optical far fields we get, see eq 3.10 of ref (12)
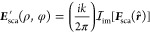
12where ***F***_sca_ is the far-field amplitude on the object side.

## Results

### BEM Simulation

We have implemented the interference microscopy features in our recently developed nanobem toolbox,^13^ with the extension for stratified media using the matrix-friendly approach of Chew.^14,20,21^ See also the Supporting Information. Although the details of the numerical implementation of the various toolbox functions are intricate, it is easy to set up and run simulations for simple geometries.

**Figure 4:**
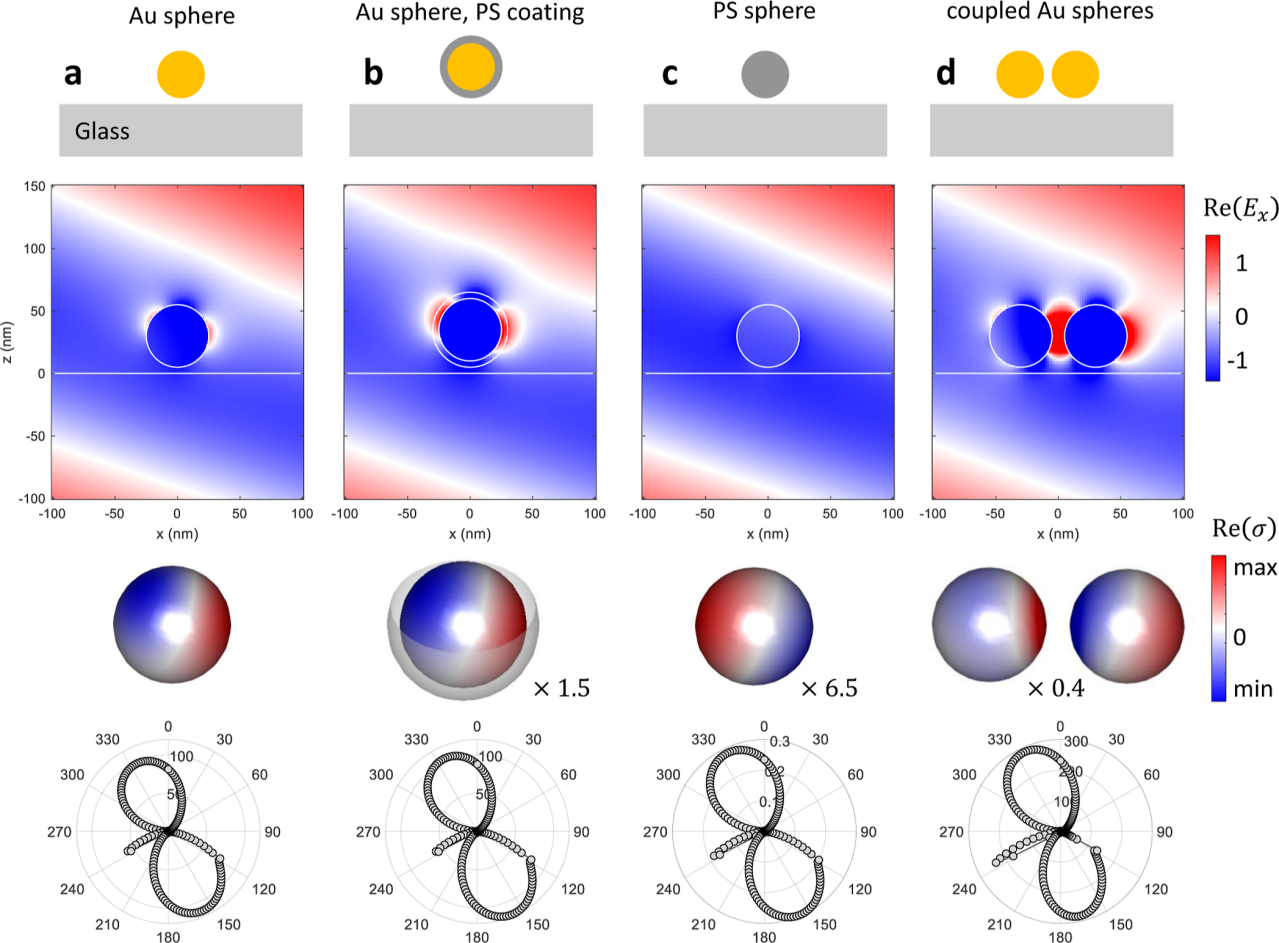
Results of bem simulations for (a) gold nanosphere (b), gold nanosphere coated with polystyrene (ps), (c) ps nanosphere, and (d) coupled gold nanospheres, embedded in water and situated 5 nm above a glass substrate. The sphere diameters are 50 nm, the coating thickness is 5 nm, and the gap distance is 10 nm. For the excitation, we use a plane wave with λ = 520 nm and tm polarization that impinges on the interface from below with an angle of −20° with respect to the *z*-axis (anticlockwise). The second row shows the real part of the electric field map *E*_*x*_(*x*, *z*) in units of the incoming field, as obtained from the nanobem toolbox [note that in panel (d), the field enhancement in the gap region is around six and exceeds the color limits], the third row shows the surface charge distributions (with scaling factors reported in the insets), and the last row shows the power scattered into a given far-field direction.

Figure 4 shows simulation results for gold (Au) and polystyrene (ps) nanospheres. Throughout, the spheres are embedded in water (refractive index *n* = 1.33) and located 5 nm above a glass substrate (*n* = 1.5). The optical properties for Au are taken from Johnson and Christy,^22^ and we use *n* = 1.59 for ps. We consider a laser excitation with a vacuum wavelength of 520 nm, where a plane wave with tm polarization impinges on the interface from below with an angle of −20° with respect to the *z*-axis (anticlockwise). In the second row of the figure, we show the electric field maps, which can be directly obtained with the nanobem toolbox functions. The computation of these maps is time-consuming because the fields have to be propagated from the sphere boundary to the field points (*x*, *z*) by means of the representation formula of eq 7. In contrast, the computation of the surface charges (third row) and the power emitted into a given far-field direction (last row) is significantly faster because these quantities can be directly obtained from the tangential boundary fields alone. Comparison of the results for metallic (Au) and dielectric (ps) nanospheres shows a phase difference of the surface charges. This is because metallic nanoparticles sustain surface plasmon resonances in the optical regime,^12^ associated with coherent electron charge oscillations at the metal surface, which oscillate close to resonance with the usual 90° phase delay with respect to the driving laser fields. A large field enhancement in the gap region between the coupled spheres is observed, which is a well-known property of surface plasmon resonances.^23^ These results demonstrate that the nanobem toolbox is well suited for the numerical solution of Maxwell’s equations for a variety of different geometries and samples.

**Figure 5:**
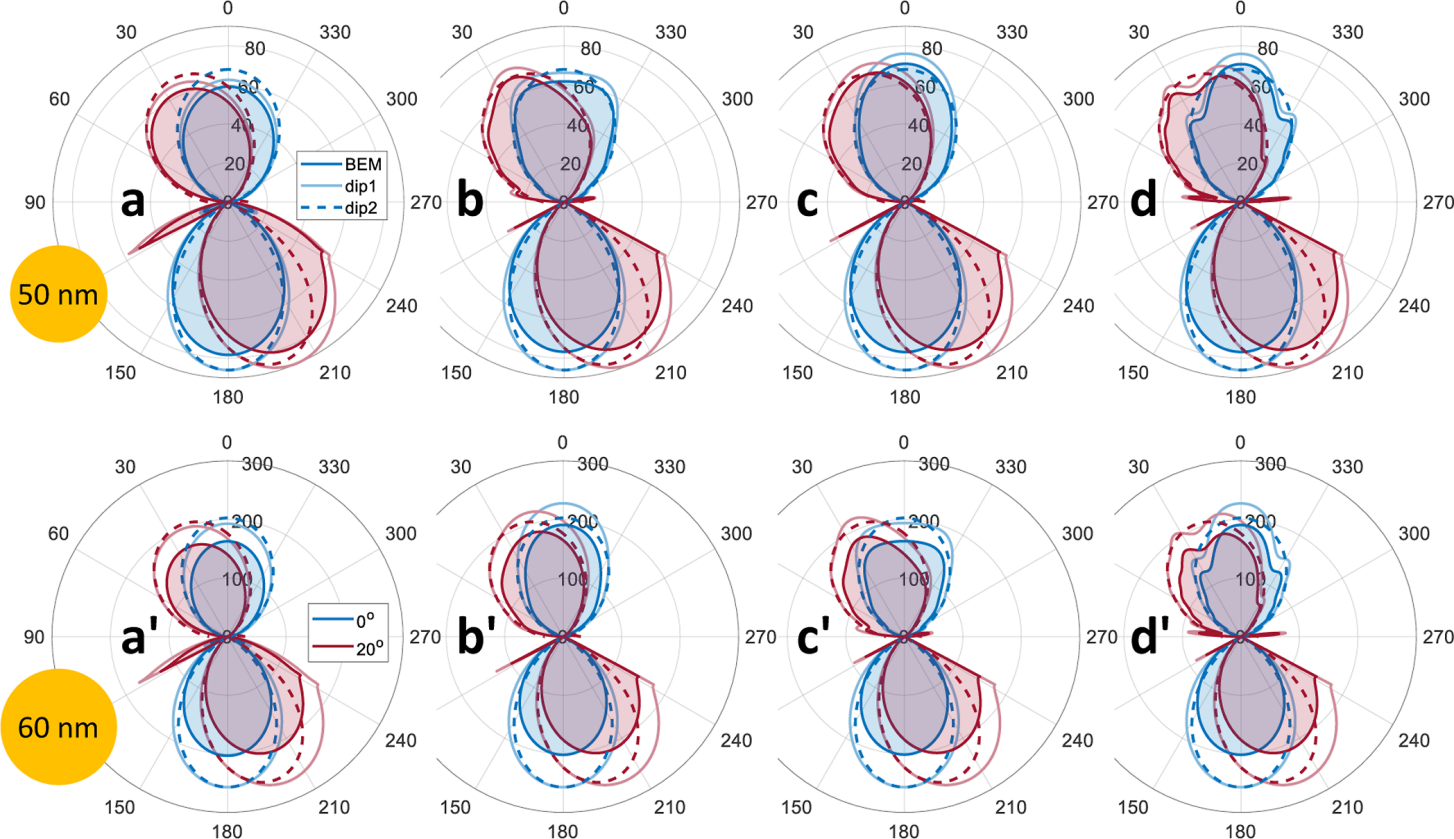
Far fields for gold spheres with different diameters and for gap distances between the sphere and glass–water interface of (a) 5 nm, (b) λ/2, (c) λ, and (d) 2 λ, with λ = 520 nm. In the simulations, a plane wave impinges on the interface from below with an angle of 0° (blue lines) and −20° (red lines). The polar plots indicate the power radiated in the far-field zone into a given direction. We compare the results of the full bem simulations (solid lines with shaded areas, BEM) with those obtained with the dipole approximation (faint solid lines, dip1), as described in more detail in the text, and a model where we consider dipole radiation in homogeneous space and consecutive diffraction of the emitted fields at the glass–water interface (dashed lines, dip2).

In Figure 5, we show the emission patterns of gold nanospheres with different diameters and distances to the substrate interface. We compare the results of the full bem simulations (solid lines with shaded areas, BEM) with those for dipole approximations assuming a polarizable particle. In the latter, the nanoparticle scattering is either approximated by the scattering of a point dipole above an interface (faint solid lines, dip1), see also eq (D.6) of ref (16), or the scattering of a point dipole in a homogeneous space, followed by the diffraction of light at the substrate interface^6^ (dashed lines, dip2). We observe that the dipole approximation works well for nanoparticle diameters smaller than 50 nm. For larger particles, retardation effects gain importance, and a full simulation approach becomes indispensable.

### Simulation of Imaging

In the previous section, we have discussed that the nanobem toolbox allows computing the incoming fields and far fields for typical interference microscopy setups without introducing any modifications. Runtimes for typical setups range from a few tens of seconds to minutes on a normal desktop computer. In what follows, we discuss the steps needed to compute the interference images, see eq 4, that can be directly compared with the experiment. In the Appendix, Numerical Implementation, we provide details about our numerical implementation of the Richards–Wolf approach.

We first discuss the simulation of the experimental setup depicted in Figure 10, where the incoming laser beam passes through a quarter wave plate (wp) and the focus lens and excites the nanoparticle, and the reflected and scattered fields are imaged. The field manipulation of the quarter wave plate is accounted for by a Jones matrix with the fast *x*-axis rotated clockwise by 45°,^19^ as discussed in more detail in the Supporting Information, both in the excitation (light propagation in the + *z*-direction) and detection (light propagation in the – *z*-direction) path. For imaging, we apply the same Jones matrix in the  function, see eq 11, that allows for field manipulations in the back focal plane.

**Figure 6:**
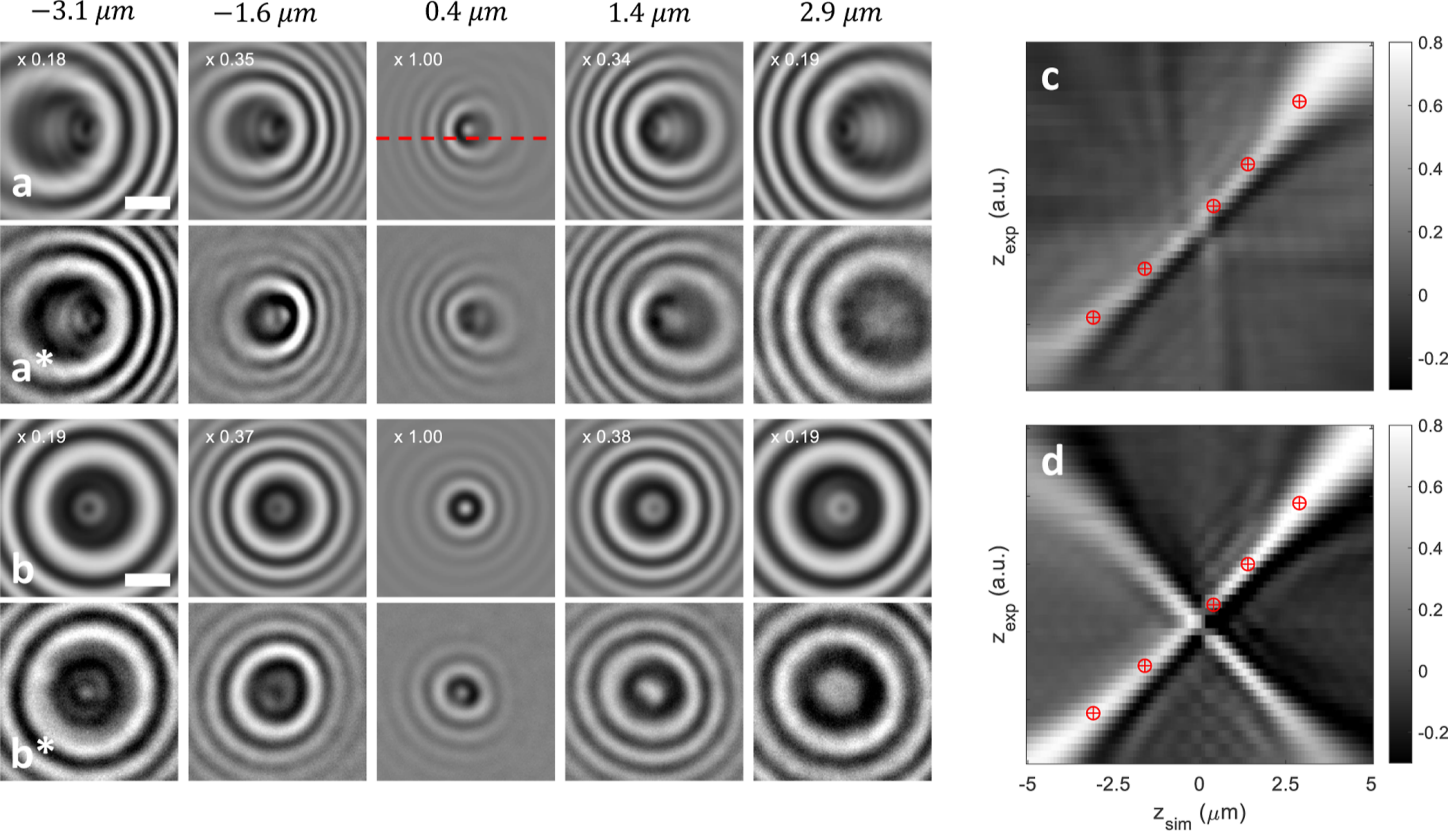
(a,b) Simulated and (a*,b*) measured iscat images for different focal plane positions reported above the panels. We use the setup depicted in Figure 4a, but for a gold nanosphere with a diameter of 55 nm, and for angles θ of the incoming plane wave with respect to the + *z*-axis of (a) 14° and (b) −2°. The scale bar is 1 μm, and the red dashed line indicates *y* = 0. (c,d) Correlation plots from which the best overlap between the simulated and measured images is obtained. For details, see text.

**Figure 7:**
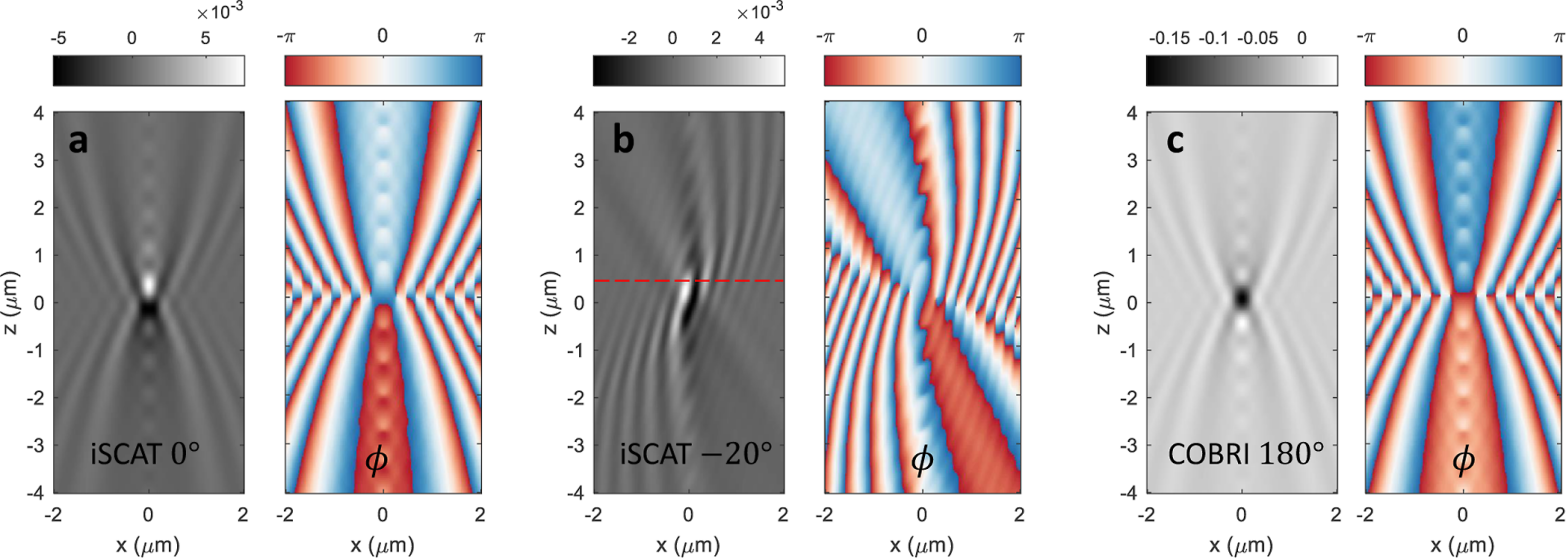
iscat, phase ϕ = arg(***E***_ref_^′^·***E***_sca_^′^*), and cobri maps for different focal positions *z*. We use the same simulation setup as in Figure 4a, and plane wave excitations with tm polarizations and incoming angles of (a) 0°, (b) −20°, and (c) 180° for cobri. Note that the plane of highest contrast, as indicated by the dashed red line, does not necessarily coincide with the focal plane of the objective.

**Figure 8:**
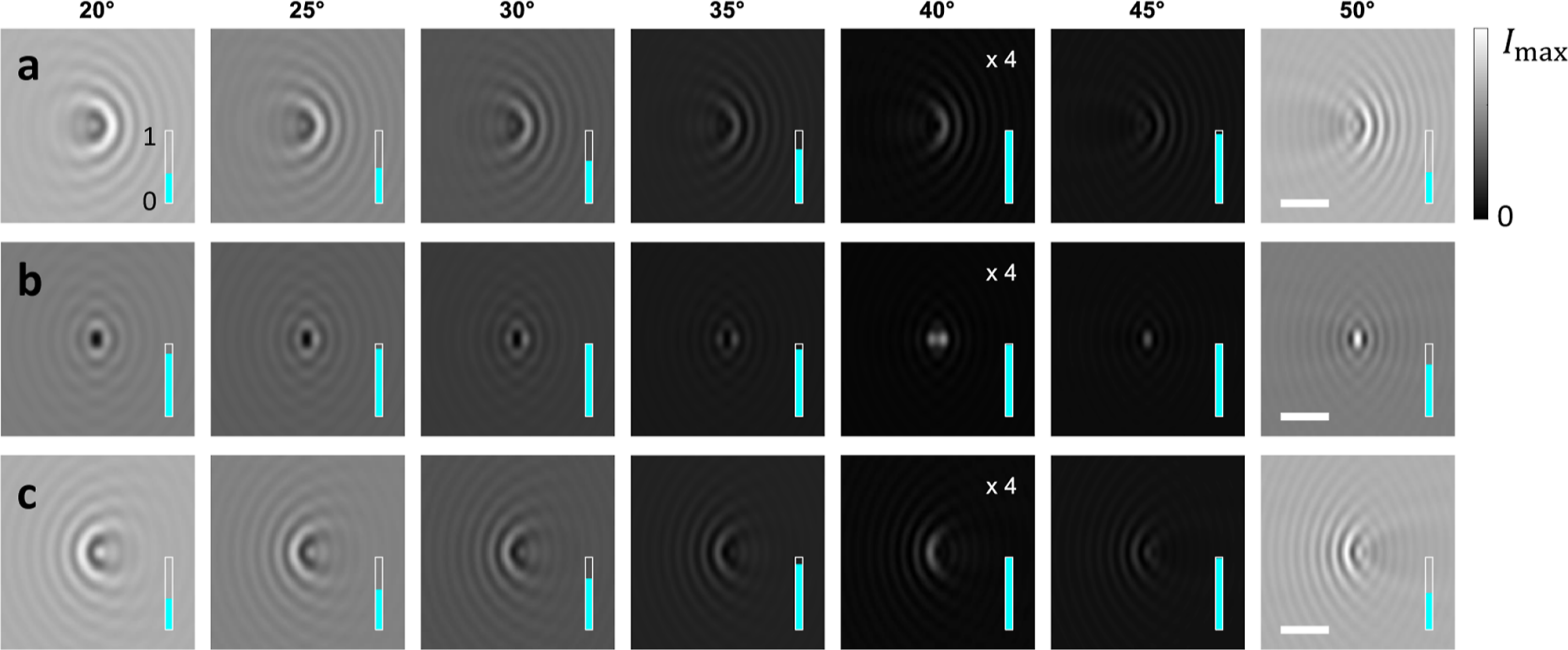
iscat maps  without background subtraction for different incoming angles (see top) around the Brewster angle θ_B_ ≈ 42°, and for a gold nanosphere with a diameter of 40 nm located 5 nm above a glass–water interface. We use focal plane positions *z*_foc_ of (a) −0.5, (b) 0, and (c) + 0.5 μm. The light blue bars inside the panels report the Michelson contrast, which is bound to values between zero and one. The scale bar is 1 μm, the iscat maps for θ = 40° have been multiplied by a factor of 4 for better visibility, and the maximal intensity *I*_max_ is the same in all panels.

**Figure 9:**
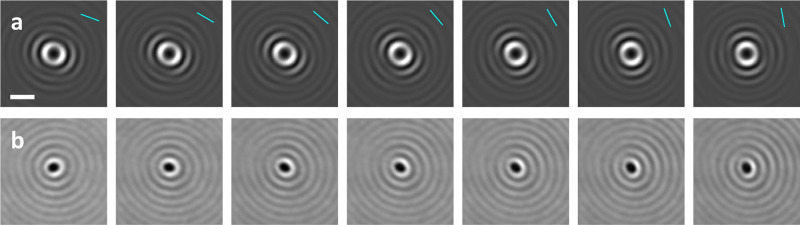
(a) Simulated and (b) experimental iscat maps for a silver nanocube located on top of a glass–air interface (focal plane at *z* = −0.4 μm). The incoming light propagates in the positive *z*-direction, and the light polarization angles φ_pol_ are indicated in the upper right corners of the panels in (a).

**Figure 10:**
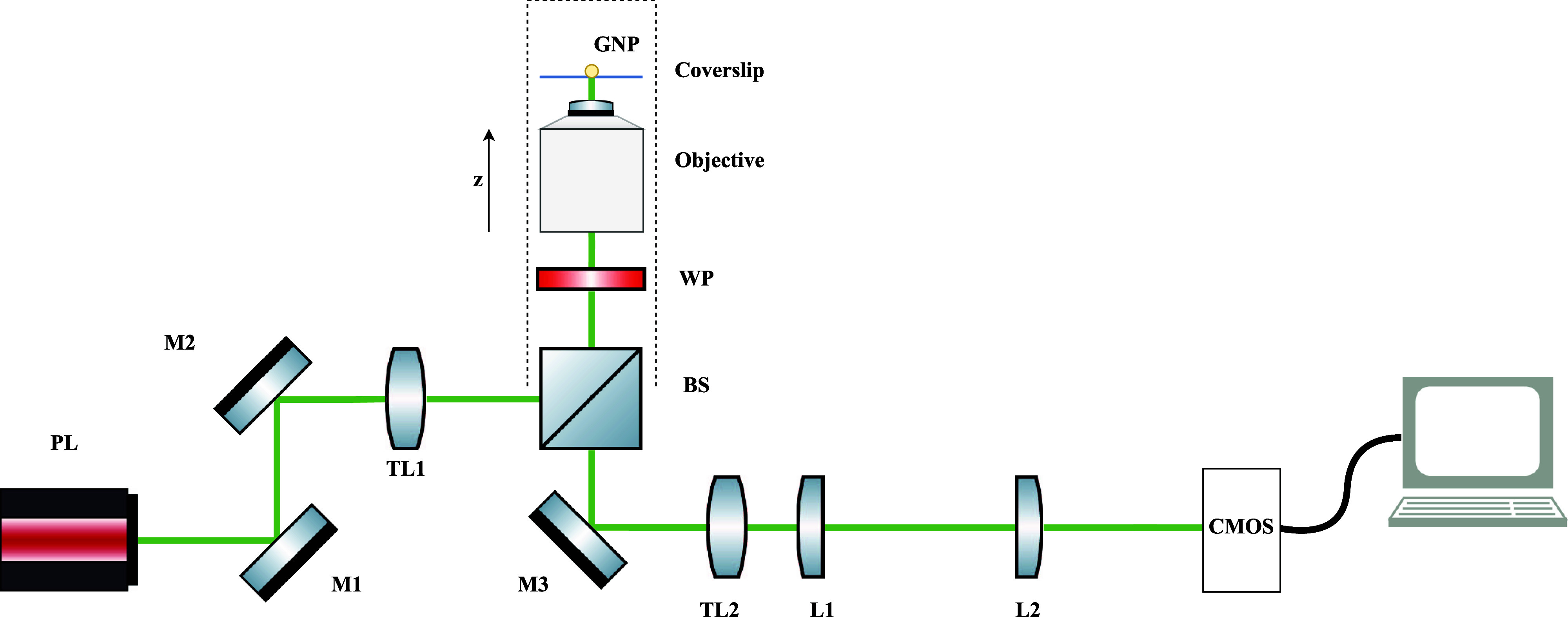
Optomechanical components used in our experiments. Silver-coated Thorlabs mirrors (M1, M2, and M3), tube lenses TL1 with focal length *f*_1_ = 165 mm, TL2, *f*_2_ = 165 mm, and lenses L1, L2 form a 4*f*-system; WP and BS are abbreviations for a waveplate and a beam splitter, respectively. For setup I: BS is a polarizing beam splitter, lenses L1, *f*_3_ = 150 mm, L2, and *f*_4_ = 500 mm. For setup II: BS is a nonpolarizing beam splitter and WP is not present, lenses L1, *f*_3_ = 75 mm, L2, and *f*_4_ = 200 mm.

Figure 6 shows (a,b) simulated iscat maps for a gold nanosphere with a diameter of 55 nm situated in air and 5 nm above a glass substrate. In the simulations, we compute the background-subtracted interference between the secondary (reflected) incoming fields and the scattered fields via
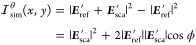
13

The angle of the incoming field with respect to the + *z*-axis is denoted with θ and ϕ(*x*, *y*) is the phase between the reference and scattered fields. For sufficiently small particles,  is the interferometric point spread function ipsf of Gholami Mahmoodabadi et al.^6^

The angle θ of the incoming plane wave is (a) 14° and (b) −2°, respectively. The different panels report the images at different focal plane positions (indicated on top) with respect to the interface position of the glass substrate at *z* = 0. We additionally obtained iscat images  for various focal plane positions *z*_exp_. For details of the experiment, see the Appendix, Experimental Setup and Parameters. Note that in the experiment, the precise value of *z*_exp_ is unknown, and a fit to theoretical results is generally needed to obtain a reliable estimate for *z*_exp_. In our work, we simulated images  for various focal plane positions *z*_sim_ and determined the experimental focal plane position *z*_exp_ from the highest correlation with the simulation results
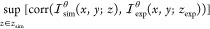
14

The correlation is computed through the matlab function corr2; the corresponding matlab files are provided in the Supporting Information. Figure 6c,d shows the correlation maps between the simulated and experimental iscat images. The red symbols indicate the highest correlation for the experimental data presented in (a*,b*). The images are in good agreement with the respective simulation results.

Interestingly, for off-axis illumination, as in a*, the correlation plot yields an unambiguous *z*_exp_ for each data set. This is not the case for the data in b*, which was recorded with on-axis illumination. Here, the correlation plot shows two lines with a crossing in the focal plane. For a single iscat image, it will thus be difficult to judge whether it is overfocused or underfocused.

Our simulations allow us to take a closer look at this behavior. When plotting the simulations similar to those in Figure 6a in the *xz*-plane for *y* = 0 (see the red dashed line), we obtain the iscat maps shown in Figure 7. A localized diffraction-limited spot when the focal plane coincides with the particle plane and characteristic interference features between the incoming and scattered waves are observed. In the colored panels of the figure, we plot the interference angle ϕ, see eq 13, as extracted from our simulations. The pronounced features in the iscat maps can be traced back to the phase variations in the interference term of eq 13. We again see that illumination at an angle leads to a clear directionality in the observed interference features, which explains the unique mapping observed in the correlation plots in Figure 6. Panel (c) shows that a similar behavior can be found in cobri. Note that in the cobri simulation for the excitation from above, we simply have to change the propagation direction of the incoming wave to 180°, resulting in a propagation into the negative *z*-direction. The rest of the simulation remains identical.

Another advantage of off-axis illumination is illustrated in Figure 8, where we simulate iscat images with an illumination angle around the Brewster angle. We simulate a gold nanosphere (diameter 55 nm) at a glass–water interface. For *x*-polarized light, no light will be reflected at the Brewster angle θ_B_. This leads to a dark-field image where ***E***_refl_^↓^ = 0 and . For small deviations from θ_B_, the magnitude of the reference light, and thus the contrast in the iscat image, can be tuned continuously. This presents an alternative to other contrast-tuning modalities commonly used in iscat, which are either based on polarization or on attenuators in the back–focal plane.^24−26^

In Figure 9, we present simulations and experiments for nonspherical particles, specifically for a silver nanocube with a side length of 100 nm. In the simulations, we use the dielectric function of ref (22) details of the experiments can be found in the Appendix, Experimental Setup and Parameters. The data were obtained with linear polarization at an angle φ_pol_ in the *xy*-plane. Clearly, a rotation of φ_pol_ also rotates . The simulations, for which only the particle shape in the bem simulation had to be changed, and experiments show good agreement. These results could not be easily attained with a simple dipole model, demonstrating the versatility of our numerical method.

### Additional Features and Limitations

Various iscat modalities can be modeled with our toolbox. A prominent example is iscat in a confocal scanning geometry, where the excitation beam is focused and scanned across the sample. Implementing this modality using our toolbox is described in the Supporting Information, where we also provide links to open-source demo files.

Another example is iscat with excitation light of limited longitudinal coherence, which prevents unwanted interference from reflections off interfaces other than the one close to the sample. This affects the interferometric point spread function. It can be simulated by calculating the ipsf for a number of wavelengths out of the spectrum of the light source. A weighted, incoherent sum of the simulated intensities will provide the final simulation result.

We briefly comment on the accuracy and the limitations of our bem approach. The Stratton-Chu approach and the Galerkin implementation are known to give accurate results for sufficiently fine particle discretizations.^27^ The error due to the boundary discretization scales with *h*^3/2^, where *h* is the mesh size. As a rule of thumb, the ratio between *h* and the effective wavelength in the medium should be at least of the order of 1:5. The nanobem toolbox in its present form works best for nanoparticle discretizations with a few thousand boundary elements, say up to 10,000 elements, whereas runtimes for structures with more elements become prohibitively long. The toolbox can currently only handle particles composed of materials with homogeneous material properties, which are separated by abrupt interfaces, but not particles with genuine inhomogeneous material properties. See also the Supporting Information for a discussion of what has to be considered by users in setting up their own simulations.

## Summary

We have developed a simulation platform for interference microscopy, such as iscat or cobri, using a generic Maxwell solver based on the boundary element method. The software, together with a detailed description, is provided in the Supporting Information. Here, we presented the main building blocks of our simulation approach and provided several examples. Experiments with gold nanospheres and silver nanocubes show good agreement with our simulation. The latter cannot be simulated with a simple dipole model. We found that iscat contrast can be increased using an excitation close to the Brewster angle, demonstrating the use of our toolbox for exploratory purposes. Altogether, we hope that our software will be a valuable tool for researchers working in this field.

## Experimental Setup and Parameters

Our experimental data were recorded from two different iscat setups, and in the following, we provide parameters and technical details of these.

Setup I is used for the data shown in Figure 6. Our light source is an ultrafast pulsed laser (Coherent Monaco) with a central wavelength λ = 1035 nm. By frequency doubling before entering the iscat setup, the resulting wavelength is λ = 517.5 nm. We use an immersion oil objective (ZEISS, EC “Plan-Neofluar” 40*x*/1.30 Oil DiC M27) with 1.3 NA. The particles are diluted with distilled water and then applied to a glass coverslip. We let the mixture of GNP and water dry for 24 h before using it. The coverslip with the nanoparticles (DNA-functionalized GNPs: Custom Oligo Conjugated Spherical Gold Nanoparticles for Zero Order Diagnostics from nanopartz, functionalized with a 21 nucleotide-long DNA sequence) on top is mounted on a MadCityLabs nanotranslation stage, which enables us to scan the particle through the focus of the objective. For the detection of the superposed fields, we use a CMOS camera (FLIR Blackfly BFSU3-50S5M). We operate with the matlab image acquisition toolbox for image acquisition. Table 2 summarizes further details.

**Table 2:** Experimental Parameters Used for Figures 6 and 9^a^

description	symbol	setup I	setup II
particle position	*z*_p_	on coverslip	on coverslip
particle material and shape		gold sphere	silver cube
particle size		55 nm diameter	100 nm cube length
propagation angles	θ	–2°, 14°	0°
wavelength	λ	517 nm	450 nm
refractive index object side	*n*	1.5	1.5
refractive index image side	*n*′	1.0	1.0
numerical aperture	NA	1.3	1.42
magnification total system	*M*_tot_	133.33	160
pixel size	pix_size_	3.45 μm	1.85 μm
field of view	FOV	3.9 × 3.9 μm^2^	4.4 × 4.4 μm^2^
polarization		circular	linear

a The propagation angles in setup I were determined by best correspondence with the simulations. The polarization is indicated for the light impinging on the particle.

Setup II is used for the data shown in Figure 9. Our light source is an LED with a wavelength λ = 450 nm. The mode is cleaned by the polarization maintaining fiber. The polarization state is then set by the fiber polarization controller (Thorlabs MPC320). It transforms the state of polarization of the laser output to any arbitrary state. A nonpolarizing 10:90 cube beam splitter is used in the setup. In this case, 10% of the laser power is sent to the sample, and 90% of the signal from the sample is transmitted to the CMOS camera (MER2-1220-32U3M, DAHENG IMAGING). No waveplate is needed for this setup. The state of polarization is measured by the polarimeter (Thorlabs PAX1000) mounted above the sample. The degree of polarization for all the measurements stayed above 90%, and ellipticity was lower than 0.4°. We use an immersion oil objective (Olympus PlanApo 60× with 1.42 NA).

The particles are 100 nm silver nanocubes (NanoXact Silver Nanocubes-PVP) from nanocomposix. 20 μg/mL are applied to the hydrophilic-treated (plasma-cleaned) coverslip for 10 min. The solution is then blown away. This procedure results in nanocubes being deposited on the glass surface. The data is acquired for a series of linear polarizations of the incoming field. For each angle of linear polarization ϕ_pol_, we record ten images with an exposure time of 7 ms. In postprocessing, their mean is calculated. The resulting images are Fourier-filtered to remove the interference pattern caused by the side reflections in the setup. The background image corresponding to each state of polarization is obtained by shifting the nanocube away from the field of view and taking the measurement. The background is then removed in postprocessing. Table 2 summarizes further details.

## Numerical Implementation

**Figure 11:**
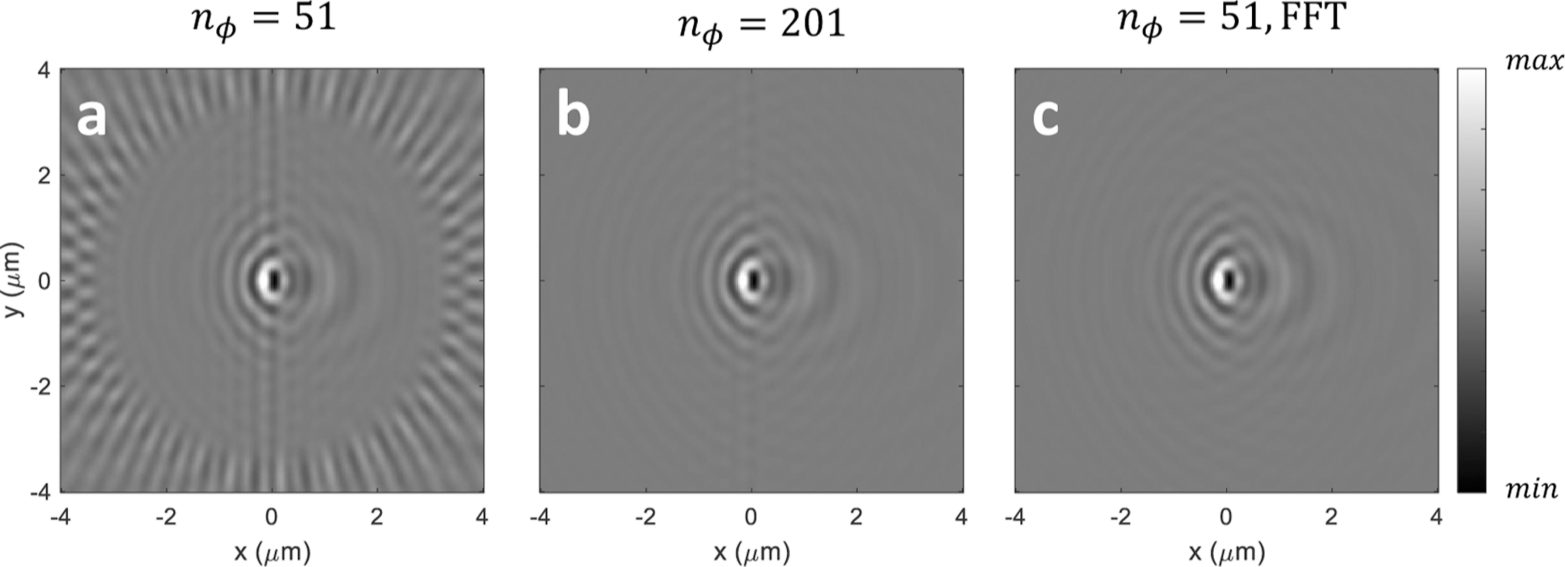
Simulation results for interference between reflected incoming and scattered fields for a lens with NA = 1.3. We use the Richards–Wolf approach and different angle discretizations; this is, *n*_θ_ = 50 and (a) *n*_ϕ_ = 51, (b) *n*_ϕ_ = 201, and (c) *n*_ϕ_ = 51 together with the integration procedure of eq 16.

In this Appendix, we provide some details about our implementation of the Richards–Wolf imaging procedure. We first note that the most time-consuming part in our simulation approach is the solution of the bem working equations. We have thus currently refrained from implementing a full fast Fourier transform (fft) in the evaluation of the Richards–Wolf integration of eq 11, see refs (12 and 18) for a more detailed discussion. In principle, such an approach would not be overly complicated and could be added to our software in the future; however, a more direct evaluation of the imaging integral in eq 11 has the advantage that the far-field (ϕ, θ) and image (ρ, φ) coordinates can be chosen independently from each other, in contrast to fft where the two coordinate systems are directly linked. Figure 11a shows the simulated iscat image for the setup depicted in Figure 4 and for an equidistant discretization for the azimuthal and polar angles. One observes the typical iscat interference features in the center but also numerical artifacts in the outer regions of the image. These artifacts can be traced back to the exponential term in eq 11, which oscillates wildly for larger values of ρ. Indeed, when increasing the number of azimuthal discretization points in panel (b), these artifacts start to disappear. A computationally more efficient approach is to perform a fft transformation for the (equally discretized) azimuthal coordinate in the far fields
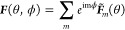
15

Each term of the Fourier series can then be integrated analytically, see eq (3.21) of ref (12)
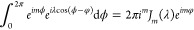
16where *J*_*m*_ is the Bessel function of order *m*. In particular, for large values of λ, eq 16 gives accurate results for a much smaller number of discretization points, see panel (c), in comparison to the direct evaluation. The evaluation can be additionally accelerated through truncation of the Fourier series, although this is not needed in most cases of interest.

## Supporting Information Available

nanobem24.zip Simulation software and demo programs for selected iscat simulations (ZIP)
nanobem-iscat24.pdf User guide with installation details for matlab toolbox for iscat simulations (PDF)
