# Supplementary material for: Unified Simulation Platform for Interference Microscopy
Source: ACS Photonics. 2024 Jun 17;11(7):2745–56. doi: 10.1021/acsphotonics.4c00621 (PMC11258784; doi:10.1021/acsphotonics.4c00621)
Supplement: Supplementary file 2 — ph4c00621_si_002.pdf [file ph4c00621_si_002.pdf]

Unified simulation platform for interference microscopy: Supplementary Information

Felix Hitzelhammer,[1,2,3] Aneka Dostlov,[4,2,3] Ilia Zykov,[2,3] Barbara Platzer,[2,3] Clara Conrad-Billroth,[2,3] Thomas Juffmann,[2,3] and Ulrich Hohenester[1,*]

[1] *Institute of Physics, University of Graz, Universitätsplatz 5, 8010 Graz, Austria*

[2] *University of Vienna, Faculty of Physics, VCQ, 1090 Vienna, Austria*

[3] *University of Vienna, Max Perutz Laboratories, Department of Structural and Computational Biology, 1030 Vienna, Austria*

[4] *Department of Optics, Faculty of Science, Palack University, 17. listopadu 12, 77900 Olomouc, Czech Republic*

In this Supplementary Information we present and discuss the main features of the `+optics` folder containing the additional classes required for the simulation of interference microscopy, as well as the various demo files for the simulation of iSCAT, COBRI, and related techniques. The software has been fully incorporated into the NANOBEM toolbox.

17 pages S1–S17

4 figures S1–S4

0 tables

* Corresponding author. E-mail `ulrich.hohenester@uni-graz.at`.

Getting started

In this section, we briefly describe how to set up the toolbox and how to run simple BEM simulations for interference microscopy. To install the toolbox, one must add the path of the main directory `nanobemdir` of the NANOBEM toolbox as well as the paths of all subdirectories to the MATLAB search path. This can be done, for instance, through

```
>> addpath(genpath(nanobemdir))
```

To set up the help pages, one must once change to the main directory of the NANOBEM toolbox and run `makehelp`

```
>> cd nanobemdir
>> makehelp
```

Once this is done, the help pages, which provide detailed information about the toolbox, are available in the MATLAB help browser under `Documentation>Supplemental Software`. See also [1, 2] for further details. In the NANOBEM toolbox provided in the Supporting Information we have included the `+optics` folder into the main directory of the toolbox. The command `addpath(genpath(nanobemdir))` then automatically makes all toolbox and `+optics` functions available. The demo programs accompanying this paper as well as a short documentation of the novel classes and functions is available in the help pages of the toolbox, see also Fig. S1. For the demo programs one simply has to run the MATLAB files.

A simple example

As a first example we consider a gold nanosphere above a glass substrate. The example closely follows the demo file `demoiscat01.m`. Each BEM simulation starts by defining the materials, the substrate or stratified medium, and the discretized particle boundary.

```
mat1 = Material( 1.50 ^ 2, 1 );               %  optical properties for glass
mat2 = Material( 1.33 ^ 2, 1 );               %  optical properties for water
mat3 = Material( epstable( 'gold.dat' ), 1 );  %  optical properties for gold
mat = [ mat1, mat2, mat3 ];                   %  unique material vector

layer = stratified.layerstructure( mat( 1 : 2 ), 0 );    %  layer structure
diameter = 50;                                           %  sphere diameter
p = trisphere( 144, diameter );                          %  discretized sphere boundary
p.verts( :, 3 ) = p.verts( :, 3 ) + 0.5 * diameter + 5;  %  shift nanosphere
tau = BoundaryEdge( mat, p, [ 3, 2 ] );                  %  boundary elements
```

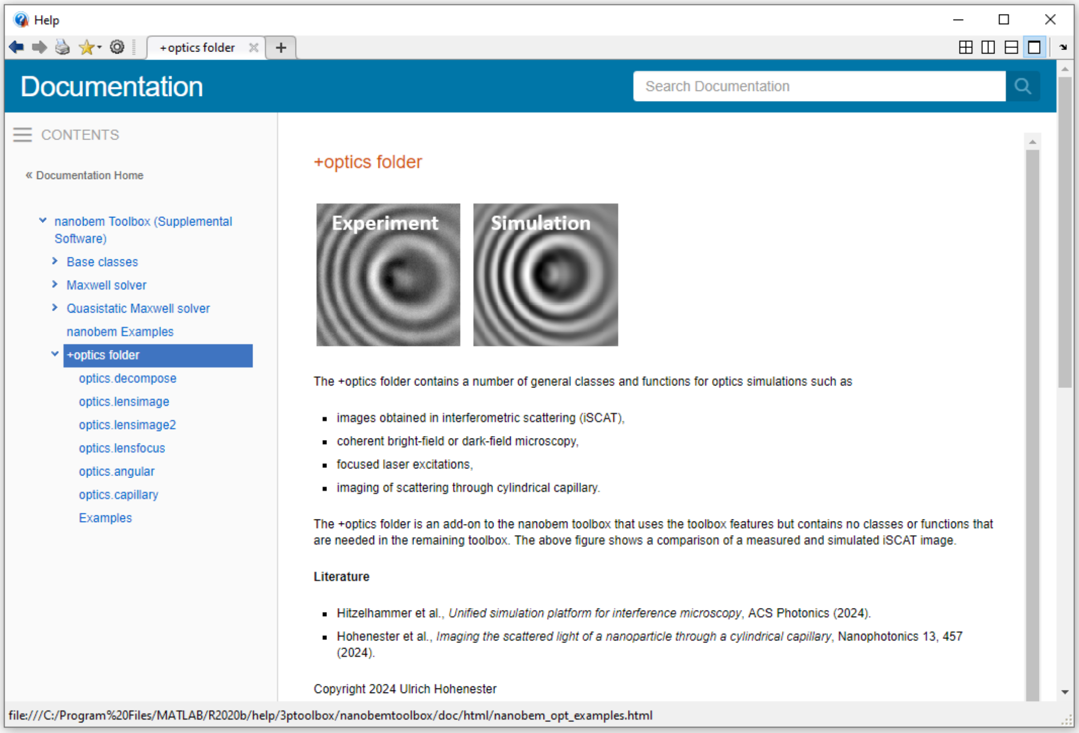

FIG. S1. Help pages of the NANOBEM toolbox which includes the `+optics` features for the simulation of coherence microscopies.

In line 4 we define a unique material vector, and in line 6 we define a layer structure with the interface at $z = 0$ and the material `mat(1)` (glass) below the interface and `mat(2)` (water) above. In line 8 we generate a discretized sphere boundary with a diameter of 50 nm, which is shifted in line 9 to a position where the lowest point of the boundary is located 5 nm above the interface at $z = 0$. Finally, in line 10 we define the boundary elements of the discretized boundary, which can be submitted to the BEM solvers provided by the NANOBEM toolbox. We are now ready to define the incoming fields and to solve the BEM equations.

```
11  k0 = 2 * pi / 520;          %  wavenumber of light in vacuum
12  pol = [ 1, 0, 0 ];          %  polarization of incoming light
13  dir = [ 0, 0, 1 ];          %  propagation direction
14  einc = optics.decompose( k0, pol, dir );  %  planewave decomposition
15  qinc = einc( tau, 'layer', layer );       %  inhomogeneities for incoming fields
16  bem = stratified.bemsolver( tau, layer, 'order', [] );  %  initialize BEM solver
17  sol = bem \ qinc;                                        %  solve BEM equations
```

In lines 11–13 we define the wavenumber of light in vacuum, and the polarization and propagation direction of the incoming light. In line 14 we set up an `optics.decompose`

object for a planewave decomposition of the incoming fields. We will comment further below on the differences between this object and the `planewave` objects provided by the toolbox. In line 15 we evaluate the inhomogeneities `qinc` of the incoming fields in presence of the layer structure. In this evaluation the secondary reflected and transmitted fields are automatically added. Finally, in line 16 we set up the BEM solver for the stratified medium and finally solve the working equations of the BEM approach, to obtain a solution object `sol` that stores the tangential electromagnetic fields at the particle boundary.

The main part of the BEM simulation is finished at this point. What remains to be done is to compute the far-fields and to submit them to imaging.

```
18 NA = 1.3;                                 %  numerical aperture
19 air = Material( 1, 1 );                   %  optical properties for air
20 rot = optics.roty( 180 );                 %  rotation matrix for imaging lens
21 lens = optics.lensimage( mat1, air, k0, NA, 'rot', rot );
22 far = farfields( sol, lens.dir );         %  farfields from BEM solution
23 refl = secondary( einc, layer, 'dir', 'down' );
```

In line 18 we define the numerical aperture of the imaging lens, in line 19 we define the material properties on the image side of the objective (air), and in line 20 we initialize a rotation matrix (180° around y-axis) that rotates the optical axis from the $+z$ to the $-z$-direction. In line 21 we set up an `optics.lensimage` object that allows us to perform the Richards-Wolf imaging procedure. When providing the rotation matrix to this object, all rotations of fields and propagation directions will be done automatically by the `lens` object. In line 22 the BEM solution and the `lens` object work together: we compute the optical far-fields $\boldsymbol{F}(\hat{\boldsymbol{r}})$ of the BEM solution that propagate in the directions $\hat{\boldsymbol{r}}$ requested by the lens object. Finally, in line 23 we compute the secondary fields of the incoming excitation that propagate downwards. For the planewave excitation from below, the secondary fields are the fields reflected at the interface. We are now ready to compute the imaged fields.

```
24 x = 2000 * linspace( -1, 1, 201 );  %  image positions
25 z = 500;                            %  focus plane
26 focus = [ 0, 0, z ];                %  focus position
27 isca = efield( lens, far,  x, x, 'focus', focus );  %  scattered image fields
28 iref = efield( lens, refl, x, x, 'focus', focus );  % incoming  image fields
29 isca = flip( isca, 1 );                   %  flip x-axis because of rotation matrix
30 iref = flip( iref, 1 );                   %  flip x-axis because of rotation matrix
31 im = dot( isca + iref, isca + iref, 3 );  %  interference image
```

In line 24 we define the image field positions and in line 25 the focus plane. In line 26 we define the focus position of the imaging system, and finally compute in lines 27, 28 the imaged fields. In this computation we essentially solve the integrals entering the Richards-Wolf approach. Finally, we have to flip the images in the x-direction because of the rotation matrix in the `lens` object. In line 30 we compute the iSCAT intensity $|\boldsymbol{E}_{\rm sca} + \boldsymbol{E}_{\rm ref}|^2$, which can be plotted and directly compared with experiment. Interested users might like to consult and run the demo programs distributed with our software.

Optics classes and functions

The additional features needed for the simulation of interference microscopy are stored in the `+optics` folder.

```
+optics.decompose       %  planewave decomposition of fields
+optics.lensimage       %  imaging of fields through lenses
+optics.lensfocus       %  focusing of incoming fields through lens
+optics.angular         %  angular spectrum representation
```

None of the functions is optimized for speed, although they are usually sufficiently fast and designed to be versatile for the simulation of a wide range of different setups. In the following we describe the various classes and functions in more detail.

optics.decompose

This class stores a planewave decomposition.

```
classdef decompose
  %  Planewave decomposition.
  properties
    k0      %  wavenumber of light in vacuum
    efield  %  electric field components
    dir     %  propagation directions
  end
end
```

`k0` is the wavenumber of light in vacuum, and `efield` and `dir` are the field amplitudes $\boldsymbol{\epsilon}$ and wavevector direction $\hat{\boldsymbol{k}}$, respectively. In case of a single plane wave `efield` and `dir` are single vectors.

```
%  plane wave with x-polarization and z-propagation
field = optics.decompose( k0, [ 1, 0, 0 ], [ 0, 0, 1 ] );
```

When `efield`, `dir` are `n×3` arrays, the electric fields are computed from

$$\boldsymbol{E}(r) = \sum_i \exp(i\boldsymbol{k}_i \cdot \boldsymbol{r})\boldsymbol{\epsilon}_i \,.$$

Note that this summation over i differs from the `planewave` objects of the NANOBEM toolbox, which allows us to obtain the response of a nanoparticle for various planewave excitations simultaneously. In contrast, with the `optics.decompose` objects we can only run simulations for a single (albeit possibly more complicated) incoming field. This functionality has certain advantages in the context of interference microscopy, in particular when it comes to the focused excitations described below. Once the object has been initialized, it can be used in different ways. The secondary fields, these are the reflected or transmitted fields of the stratified medium, can be computed from

```
field2 = secondary( fields, layer, 'dir', dir );
```

Here `layer` is the layer structure of the stratified medium, and the propagation direction must be chosen as either `'up'` or `'down'`. To evaluate the fields at given positions `pos`, we first have to place the position points in the photonic environment before submitting them to the `fields` function. For a homogeneous medium this can be done with

```
pts = Point( tau, pos );          %  place position(s) in photonic environment
[ e, h ] = fields( field, pts );  %  evaluate planewave decomposition
```

For a stratified medium the code has to be changed to

```
pts = stratified.Point( layer, tau, pos );
[ e, h ] = fields( field, pts, 'layer', layer );
```

Note that in this evaluation all secondary fields originating from reflections or transmissions at the interfaces of the stratified medium are added automatically. The inhomogeneity for the BEM solver can be computed from

```
qinc = eval( field, tau );                    %  homogeneous embedding medium
qinc = eval( field, tau, 'layer', layer );    %  particle in stratified medium
```

optics.lensimage

This class allows to simulate imaging of far-fields and planewave decompositions using the Richards-Wolf approach.

```
classdef lensimage
  %  Imaging lens, Hohenester "Nano and Quantum Optics", Eq. (3.10).
  properties
    mat1       %  material properties on object side
    mat2       %  material properties on image  side
    k0         %  wavenumber of light in vacuum
    NA         %  numerical aperture
    dir        %  directions for far-fields
    mcut       %  cutoff for angular degree
    rot        %  rotation matrix for optical axis
    backfocal  %  field manipulation in backfocal plane
  end
end
```

The class can be initialized through

```
lens = optics.lensimage( mat1, mat2, k0, NA,  ...
          'rot', rot, 'backfocal', fun, 'nphi' nphi, 'ntheta', ntheta, 'mcut', mcut );
```

The properties in the second line are optional. If they are not specified by the user, the default values are used. The meaning of `mat1`, `mat2`, `k0` and `NA` is self-explanatory. `rot` is an additional rotation matrix that allows rotating the optical axis from the $+z$-direction into other directions, as described in more detail below. `backfocal` is a user-defined function that can be used to manipulate the electromagnetic fields in the backfocal plane, for instance to mimic the effect of a quarter-wave plate. `nphi=51` and `ntheta=50` are the discretizations of the Gaussian reference sphere used in the Richards-Wolf approach, and `mcut` allows for an optional truncation of the partial Fourier transform of the optical far-fields, as discussed in the main text. Upon initialization, `lens.dir` defines the (rotated) directions in which the optical far-fields have to be computed. With a solution `sol` of the BEM solver provided by the toolbox, these far-fields can be computed and plotted through

```
far = farfields( sol, lens.dir );  %  compute farfields in directions lens.dir
plot( lens, far );                 %  plot farfields on Gaussian reference sphere
```

Once the far-field `far` or planewave decompositions `ref` of the secondary incoming fields are at hand, they can be submitted to the imaging of the Richards-Wolf approach through

```
isca = efield( lens, far, x, y, 'focus', focus );  %  imaging of farfields
iref = efield( lens, ref, x, y, 'focus', focus );  %  imaging of planewave decomposition
```

Here `x`, `y` are the image positions, and `focus` is the position of the focus spot, as will be described in more detail further below. `isca` and `iref` are arrays of size `nx`×`ny`×`3`.

optics.lensfocus

This class allows to simulate focusing of an incoming laser field, see Eq. (3.13) of [3]. The class works closely together with `optics.decompose` objects for planewave decompositions.

```
classdef lensfocus
  %  Imaging lenses, Hohenester "Nano and Quantum Optics", Eq. (3.13).
  properties
    mat     %  material properties on focus side
    k0      %  wavenumber of light in vacuum
    NA      %  numerical aperture
    rot     %  rotation matrix for optical axis
  end
end
```

The class can be initialized through

```
lens = optics.lensfocus( mat, k0, NA, 'rot', rot 'nphi' nphi, 'ntheta', ntheta );
```

`mat` is the material on the focus side where the fields are computed, `k0` is the wavenumber of light in vacuum, and `NA` is the numerical aperture. Users can also provide a rotation matrix `rot` that rotates the focused fields. The optional parameters `nphi=31` and `ntheta=30` control the discretization of the Gaussian reference sphere that is used to simulate focusing. After initialization, the user must provide the incoming fields impinging on the Gaussian reference sphere (with unit radius, the cutoff radius for a given NA is `lens.rad`) at the positions

```
[ x, y ] = deal( lens.x, lens.y );            %  Cartesian coordinates
[ phi, rho ] = deal( lens.phi, lens.rho );    %  polar coordinates
```

For instance, for an incoming Gauss-Laguerre beam with a topological charge of $m = 1$ and x-polarization we use

```
e = normpdf( lens.rho, 0, 0.5 ) .* exp( 1i * lens.phi );
e = e( : ) * [ 1, 0, 0 ];
```

Once the fields impinging on the Gaussian reference sphere are determined, the focused fields are obtained from

```
field = eval( lens, e );
field = eval( lens, e, 'focus', focus );  %  user-defined focus position in medium
```

`field` is an `optics.decompose` object that can be evaluated (for unbounded and stratified media) as previously described. We have refrained from analytic integrations for the azimuthal angle, see Eq. (3.21) of [3]. This makes the programs probably slower and less accurate, users might like to play with `nphi`, `ntheta` that control the degree of discretization of the reference sphere. On the other hand, the present implementation is relatively flexible and allows for the simulation of a wealth of focused excitations.

If needed, the fields of a planewave decomposition can be rotated by defining a rotation matrix `rot` and applying it to the `optics.decompose` object through

```
field = rot * field;     %  apply rotation matrix from lhs
field = field * rot .';  %  apply rotation matrix from rhs
```

To shift the fields by a vector `pos`, we have to compute the shift phase and multiply them to the planewave decomposition (`k` is the wavenumber in the medium)

```
shift = exp( - 1i * k * field.dir * pos .' );   %  shift phases
field = field .* shift;                         %  multiply fields with shift phases
```

Alternatively, we can use the `transform` function

```
obj = transform( obj, 'rot', rot );
obj = transform( obj, 'shift', pos, 'mat', mat );
```

to rotate the fields or shift them by the vector `shift` in a medium with the material properties `mat`. Note that in most cases of interest the rotation and shift operations are performed automatically in the `optics.lensimage` and `object.lensfocus` objects.

optics.angular

This function allows us to perform an angular spectrum decomposition, see Eq. (3.8) of [3], and is mainly implemented for testing purposes. Suppose that we know the fields `e(x,y,1:3)` in a plane `z` at the positions of a rectangular grid `(x,y)`. We assume that the fields are localized inside the grid `(x,y)` and are approximately zero outside the grid. We can then compute the far-fields using the angular spectrum representation,

```
far = angular( mat, k0, dir, e, x, y, z, 'nmax', nmax );
```

`mat` holds the material parameters of the embedding medium, `k0` is the wavenumber in free space, and `dir` are the propagation directions of the far-fields $\boldsymbol{F}(\hat{\boldsymbol{r}})$. `z=0` is the plane where the fields have been computed. The function might require a lot of memory and the computation is broken up into a loop where in each iteration `nmax=1000` directions are computed at a time. This value should suffice for most cases of interest, but can be reduced if memory becomes an issue. The output of the function are the far-fields `far` with propagation directions `dir`.

Demo programs

We provide a few demo programs that demonstrate the working principle of the software.

demooptics01.m Focusing of incoming laser beam for user-defined focus position and rotated optical axis.

demooptics02.m Imaging of point dipoles for different focus planes and for imaging system with optical axis along $+z$, see Fig. S4(a–c).

demooptics03.m Imaging of point dipoles for different focus planes and for imaging system with optical axis along $-z$, see Fig. S4(a*–c*).

demoiscat01.m Computes the iSCAT images for a gold nanosphere (50 nm diameter, embedded in water) situated 5 nm above a glass substrate. By changing the numbers in the `switch` block at the beginning of the program one can simulate different setups, this is (2) coated, (3) dielectric, and (4) coupled nanospheres.

demoiscat02.m Same as `demoiscat01.m` for single gold nanosphere and varying focus planes.

demoiscat03.m Same as `demoiscat02.m`, we additonally compare with the results of a simplified dipole model.

demoiscat04.m Same as `demoiscat02.m` but with a quarter-wave plate in the excitation and detection path.

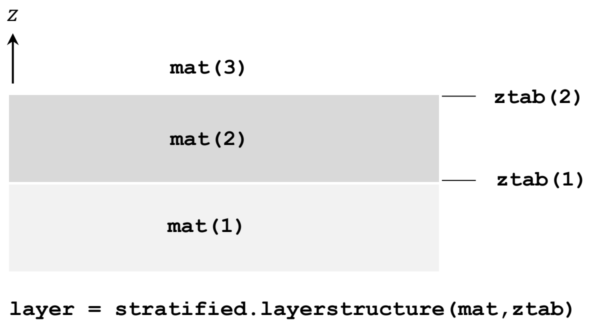

FIG. S2. Definition of layer structure with material vector `mat` and interface positions `ztab`.

demoiscat05.m Compute iSCAT images for different focal plane positions and display images together with an interactive slider.

demoiscat06.m Compute iSCAT images for comparison with Mahmoodabadi et al., Opt. Express 28, 25969 (2020), Fig. 3(b).

demofield01.m Computes the electric field maps for the different setups described above.

democorr01.m Example of how to obtain the optimal focus plane for an experimental image. In this demo file we also show how to account for a quarter-wave plate.

demofocus01.m Compute the focus fields for an incoming Laguerre-Gauss beam. We also image the focus fields using either the planewave decomposition or the far-fields obtained from the angular spectrum representation. Users might like to compare the energy transported by the waves through the focus (`P1`) and image (`P2`) plane, which should be the same apart from numerical inaccuracies.

Short list of How-tos

In this section we discuss a few how-tos that might help users to set up their own iSCAT simulations.

How to define rotation matrices? MATLAB provides a few commands for setting up rotation matrices, as needed for the rotation of particles or the optical axis of lenses. These include `eul2rotm`, `rotx`, `roty`, and `rotz`. Additionally, we provide the three functions

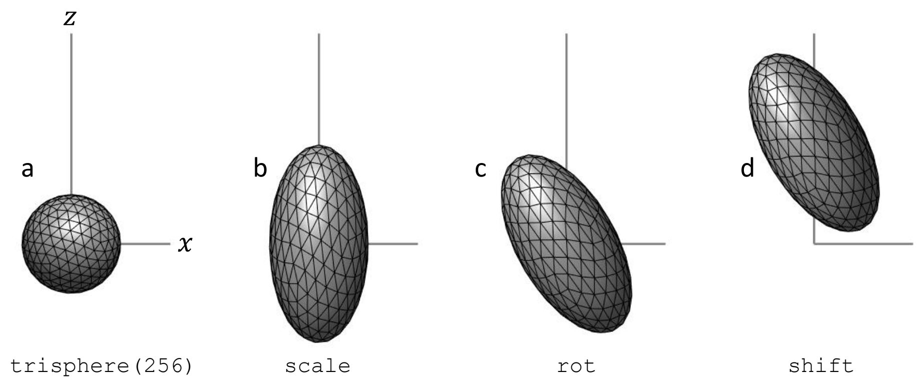

FIG. S3. Nanoparticle boundary for (a) sphere and (b–d) transformed boundaries.

```
rot = optics.rotx( t );   %  rotation around x-axis by angle t (deg)
rot = optics.roty( t );   %  rotation around y-axis by angle t (deg)
rot = optics.rotz( t );   %  rotation around z-axis by angle t (deg)
```

How to set up layer systems? To set up a layer structure in the NANOBEM toolbox, we have to define the material properties of the different media (in ascending order with respect to z) together with the interface positions (in ascending order with respect to z). See also Fig. S2. We recommend using a unique material vector including all possible materials used in the simulations, with the first n entries corresponding to the materials of the stratified medium with n layers. The remaining material entries are then used for the nanoparticles, as discussed above. The stratified medium is then set up with

```
layer = stratified.layerstructure( mat( 1 : n ), ztab( 1 : n - 1 ) );
```

How to set up particles? The NANOBEM toolbox provides a number of functions for defining the nanoparticle boundaries. As an example, in Fig. S3(a) we show a nanosphere with a given diameter that is produced with

```
p = trisphere( 256, diameter );
```

The nanoparticle boundary can then be transformed using the functions

```
1  p = transform( p, 'scale', [ 1, 1, 2 ] );
2  p = transform( p, 'rot', optics.roty( 30 ) );
3  p = transform( p, 'shift', [ 0, 0, - min( p.verts( :, 3 ) ) + gap ] );
```

In line 1 we scale the particle along the three axes of the Cartesian coordinate system, in line 2 we rotate the particle by providing a rotation matrix, and in line 3 we shift the particle to a position where the lowest point of the boundary is located a distance `gap` above the plane

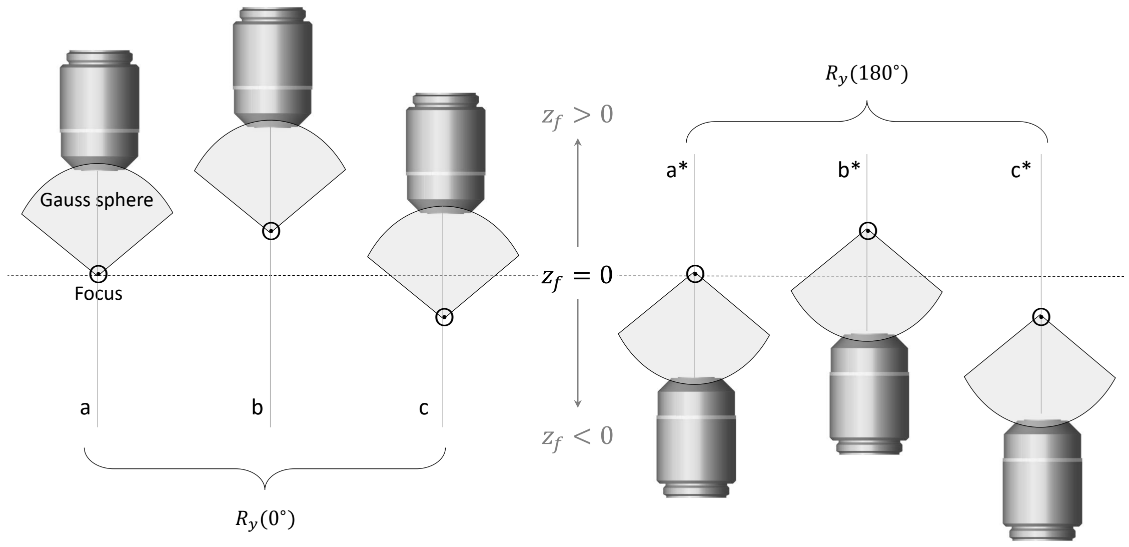

FIG. S4. Rotation of optical axis and user-defined focus position `[0,0,zf]` for imaging lenses.

$z = 0$. With the latter command one can place nanoparticles above or below the interfaces of a stratified medium. Rather than using the `transform` commands one can also directly manipulate the vertices of the `particle` object.

```
1 p.verts = p.verts .* [ 1, 1, 2 ];
2 p.verts = p.verts * optics.roty( 30 ) .';
3 p.verts = p.verts + [ 0, 0, - min( p.verts( :, 3 ) ) + gap ];
```

Note that in line 2 we use the transpose of the rotation matrix because we multiply it from the right-hand side on the vertices array `p.verts(:,1:3)`.

How to define the position and orientation of lens objects? On default the optical axis of the `optics.lensimage` object points in the $+z$-direction and the focus point is located at `[0,0,0]`. In order to rotate the optical axis we can specify in the initialization a rotation matrix `rot`,

```
lens = optics.lensimage( mat1, mat2, k0, NA, 'rot', rot );
```

To set a user-defined focus point (defined with respect to the center of the coordinate system of the BEM simulations), we can provide the position of the focus point `focus` in the evaluation of the image fields

```
im = efield( lens, field, x, x, 'focus', focus ); % user-defined focus position
```

This command works for both far-fields and planewave decompositions. Through this command the focus point is moved to position `focus` of the BEM coordinate system, as shown

in Fig. S4 for `focus=[0,0,zf]`.

A similar procedure can be used for the focusing lens. In the initialization we can pass a rotation matrix `rot` and in the evaluation the focus position `focus`, ,

```
lens = optics.lensfocus( mat1, mat2, k0, NA, 'rot', rot );
field = eval( lens, efield, 'focus', focus );
```

How to manipulate fields in the backfocal plane? Sometimes one might like to manipulate the fields in the backfocal plane of the imaging system, e.g. to mimic the effect of a quarter-wave plate. We provide a function that gives the Jones matrix for a quarter-wave plate

```
quarter = optics.quarterplate( t );  %  rotation angle in degrees
```

`quarter` is a 3×3 matrix for the optical axis along z and `t` is the rotation angle of the plate in degrees. With `optics.quarterplate(45)` we then get a quarter-wave plate that transforms an incoming field with linear polarization along x into a field with circular polarization. To modify the fields in the backfocal plane, we must provide a user-defined function `fun` that manipulates the fields according to `field=fun(field)`. For the Jones matrix `quarter` and the imaging lens object `lens` we then set

```
lens.backfocal = @( field ) field * quarter .';
```

Once the function is set, the field manipulations in the backfocal plane are performed automatically. Note that we use the transpose of the Jones matrix because we apply the matrix from the right-hand side on the array `field(:,1:3)`.

To apply the same transformations for a focusing lens `lens`, we can use the same function to manipulate the incoming fields before crossing the Gaussian reference sphere,

```
efield = fun( efield );        %  apply transformation to fields before Gauss sphere
field = eval( lens, efield );  %  focused fields
```

How to manipulate a single plane wave? To rotate, shift, and transform a plane wave we can proceed as follows:

```
1 pol = fun( pol );
2 field = optics.decompose( pol, dir );
3 field = transform( field, 'rot', rot );
4 field = transform( field, 'shift', shift, 'mat', mat );
```

In line 1 we apply a transformation function to the field component of the plane wave, which is initialized in line 2. In line 3 we rotate the fields and in line 4 we propagate the fields by the vector `shift` in a medium with material properties `mat`.

Frequently asked questions

Which units does the toolbox use?

The NANOBEM toolbox uses nanometers for length and the free-space wavenumber $k_0 = \frac{2\pi}{\lambda}$ for frequencies. The electric field is given in units of the incoming fields, the magnetic field is given in units of $Z_0\boldsymbol{H}$, where Z_0 is the free-space impedance, such that $\boldsymbol{E}$ and $Z_0\boldsymbol{H}$ have the same dimensions.

Which coordinate systems are used by the toolbox?

1. In the coordinate system of the NANOBEM toolbox one specifies the nanoparticle boundaries with respect to a user-defined origin, which is usually chosen such that $z = 0$ corresponds to the substrate interface.
2. In the coordinate system of the `optics.lensimage` object the optical axis is parallel to the z-axis and the the origin of the coordinate system is the focus point of the first Gaussian reference sphere.
3. In the coordinate system of the `optics.lensfocus` object the optical axis is parallel to the z-axis and the the origin of the coordinate system is the focus point of the Gaussian reference sphere.

To align the coordinate systems we can rotate the optical axis and define the focus position `focus` using the procedures discussed above.

How to compute optical forces?

The computation of optical forces through Maxwell's stress tensor is implemented in the toolbox. For a BEM solution object `sol` one obtains the optical force `ftot` and torque `ntot` through

```
[ ftot, ntot, frc ] = optforce( sol );
[ ftot, ntot, frc ] = optforce( sol, 'imat', imat, 'ind', ind, );
```

`imat=1` is the index to the material in which the nanoparticle is embedded, and `ind` is an optional index for selected boundary elements. `frc` is the force component for each boundary element.

How to choose the best discretization?

This is a difficult issue. Quite generally, within our BEM implementation it is guaran-

teed that the computational solution converges to the true solution for sufficiently fine meshes, and also particles with sharp edges or directly placed on interfaces of a stratified medium can be safely simulated. It is usually a good idea to run simulations with an increasing number of boundary elements and to inspect the simulation results. The final choice of fast runtimes (coarse discretizations) versus highly accurate results (fine discretizations) is left to the users, although in many cases even coarse discretizations will already give sufficiently accurate results.

How to speed up simulations for multilayer systems?

For stratified media with more than one interface the simulations can be sometimes very slow. This is usually because of an inefficient evaluation of the Sommerfeld integrals, see discussion in [2]. It often helps to use in the initialization of the BEM solver the options

```
op = odeset( 'AbsTol', 1e-5, 'InitialStep', 1e-3 );
bem = stratified.bemsolver( tau, layer, 'op', op, 'semi1', 10 );
```

Users are advised to play with the value of `semi1` to find the best performance of the code. `AbsTol` controls the accuracy of the MATLAB `ode45` integration, however, the value should not be chosen too small because otherwise the simulations get inaccurate.

How to speed up simulations for many particle positions?

To run simulations for various particle positions one has to introduce a loop over the different positions, set up the discretized particle boundary and the BEM solver, and compute the far-fields for each position separately. This can result in long simulation times. If the particle is sufficiently far away from the interfaces of the stratified medium and nearfield couplings play a minor role, there exists a more simple but approximate approach. We solve the BEM equations for a homogeneous medium and account for the shifting of the particle by evaluating the incoming fields at the shifted particle positions. The solution object is then converted to a `stratified.solution` vector, which can be used in the same manner as previously described.

```
bem = galerkin.bemsolver( tau, 'order', [] );  %  BEM solver for homogeneous medium
einc = optics.decompose( k0, pol, dir );       %  primary excitation

for iz = 1 : numel( zout )
  qinc = einc( tau, 'layer', layer, 'shift', [ 0, 0, zout( iz ) ] );
```

```
6    [ sol, bem ] = solve( bem, qinc );          %  SOL w/o nearfield couplings
7    sol = stratified.solution( qinc.tau, k0, sol.e, sol.h );
8    sol.layer = layer;                          %  approximate SOL for layer
9    ...
10 end
```

In line 5 we evaluate the incoming fields at the shifted particle position, where `zout` is an array of the vertical shift positions. Note that `qinc.tau` is an array of the shifted boundary elements. With the calling sequence in line 6 we ensure that the BEM solver is evaluated only once. Quite generally, this approach is much faster and justified when nearfield couplings of the particle to the stratified medium are of minor importance. Whether this is the case should be examined for a few shift positions through comparison with the results of the full simulations.

What can go wrong in simulation?

We have done our best to make the NANOBEM toolbox as stable as possible. Nevertheless, a number of things might go wrong. We advise users to carefully check whether the boundaries of the nanoparticles are defined properly, in particular with respect to the orientation of the boundary and the choice of inner and outer materials. For stratified media one should be also careful about the reflected Green's functions, which can be plotted for testing as described in [2]. In case of other unforeseen errors, contact one of the authors with a detailed description of the problem.

[1] U. Hohenester, N. Reichelt, and G. Unger, Nanophotonic resonance modes with the nanobem toolbox, Comp. Phys. Commun. **276**, 108337 (2022).

[2] U. Hohenester, Nanophotonic resonators in stratified media with the nanobem toolbox, Comp. Phys. Commun. **294**, 108949 (2024).

[3] U. Hohenester, *Nano and Quantum Optics* (Springer, Cham, Switzerland, 2020).
